# Barriers to the quality delivery of seasonal malaria chemoprevention in Chad and Burkina Faso: a qualitative exploration of caregivers and community distributors’ perspectives

**DOI:** 10.1186/s12936-024-05034-6

**Published:** 2024-07-19

**Authors:** Kévin Lasmi, Kelly Elimian, Laura Donovan, Narcisse Tounaikok, Adama Traoré, Tinne Gils, Christian Rassi, Madeleine Marasciulo, Sol Richardson, Gauthier Tougri, Mahamat Saleh Issakha Diar, Kevin Baker

**Affiliations:** 1 Department of Global Public Health, Karolinska Institute, Stockholm, Sweden; 2 Malaria Consortium, London, UK; 3 Malaria Consortium, Chad Country Office, N’Djamena, Chad; 4 Malaria Consortium, Burkina Faso Country Office, Ouagadougou, Burkina Faso; 5 Department of Clinical Sciences, Institute of Tropical Medicine, Antwerp, Belgium; 6 Global Health Institute, University of Antwerp, Wilrijk, Belgium; 7 Malaria Consortium US, Raleigh, NC USA; 8 Vanke School of Public Health, Tsinghua University, Beijing, China; 9 Programme national de lutte contre le paludisme, Ouagadougou, Burkina Faso; 10 Programme national de lutte contre le paludisme, N’Djamena, Chad

**Keywords:** Seasonal malaria chemoprevention, Quality of care, Caregivers, Community distributors, Qualitative study, Quality standards, Malaria prevention, Chimioprévention du paludisme saisonnier, qualité des soins, parents/tuteurs, distributeurs communautaires, étude qualitative, normes de qualité, prévention du paludisme, Quimioprevenção sazonal da malária, qualidade dos cuidados, cuidadores, distribuidores comunitários, estudo qualitativo, normas de qualidade, prevenção da malaria

## Abstract

**Background:**

Recommended since 2012 by the World Health Organization (WHO), seasonal malaria chemoprevention (SMC) is a community-based intervention to prevent malaria in children in African regions where malaria transmission follows a seasonal pattern. Following the publication of consolidated WHO guidelines for malaria, SMC is expected to reach more children in new geographies in future years. Though SMC has been shown to reduce malaria-related morbidity and mortality, there is potential for quality improvement of the intervention implementation. Assisted by ten quality standards from a framework developed by Malaria Consortium, this paper aims to better understand the quality of SMC implementation and identify potential barriers to quality delivery of SMC.

**Methods:**

A qualitative thematic analysis on data collected after the annual SMC rounds implemented in Burkina Faso and Chad in 2019 was conducted. Sixteen focus group discussions conducted with caregivers and community distributors were analysed. Three selected quality standards for SMC delivery; planning and enumeration; community engagement; and administration of SMC medicines provided overarching quality themes under which subthemes were identified.

**Results:**

Eight subthemes relating to the three quality standards were identified. Although SMC was well accepted by communities in both settings, common barriers to the quality delivery of SMC were identified including difficulty ensuring adherence to the SMC administration protocol; difficulties reaching mobile populations; concerns around adverse drug reactions; rumours, and concerns about SMC safety; and community distributors’ working conditions. Context-specific barriers included: the suboptimal timeliness of the SMC round in Burkina Faso, and the lack of involvement of female caregivers in mobilization activities in Chad.

**Conclusion:**

In the context of increased adoption of SMC, this paper provides relevant insights and recommendations for the improved implementation of SMC programmes. These include the integration of strategies addressing communities’ concerns around adverse drug reactions, gender-specific mobilization strategies, and attention to community distributors’ working conditions. It also highlights the importance and utility of further, robust research on the quality of SMC delivery.

## Résumé en français

**Background:**

Recommandée depuis 2012 par l’Organisation mondiale de la santé (OMS), la chimioprévention du paludisme saisonnier (CPS) est une intervention communautaire visant à prévenir le paludisme chez les enfants dans les régions d’Afrique où la transmission du paludisme suit un schéma saisonnier. Suite à la publication des lignes directrices consolidées de l’OMS sur le paludisme, la CPS devrait toucher davantage d’enfants dans de nouvelles zones géographiques dans les années à venir. Bien qu’il ait été démontré que la CPS réduisait la morbidité et la mortalité liées au paludisme, il y a du potentiel pour améliorer la qualité de l’implémentation l’intervention. En s’appuyant sur un cadre de normes de qualité de la CPS développé par le Malaria Consortium, cette publication vise à mieux comprendre la qualité de la mise en œuvre de la CPS et à identifier les obstacles potentiels à la qualité de la mise en œuvre de la CPS.

**Methods:**

Une étude qualitative basée sur l'analyse secondaire des données collectées après les tournées annuelles du SMC mises en œuvre au Burkina Faso et au Tchad en 2019 a été menée. Une analyse thématique de 16 discussions de groupe menées avec des parents/tuteurs et des distributeurs communautaires a été faite. Trois des éléments clés du cadre des normes de qualité pour le déploiement de la CPS ont fourni les thèmes de qualité principaux sous lesquels les sous-thèmes identifiés ont été placés.

**Résultats:**

Huit sous-thèmes relatifs aux normes de qualité ; la planification, la sensibilisation et l'engagement des communautés ainsi que l'administration des médicaments de la CPS ont été identifiés. Bien que la CPS ait été bien acceptée par les communautés dans les deux contextes, des obstacles communs à la qualité du déploiement de la CPS ont été identifiés, notamment : la difficulté d'assurer le respect du protocole d'administration de la CPS; atteindre les populations mobiles ; les préoccupations concernant les effets indésirables des médicaments ; les rumeurs et les préoccupations concernant le SMC; et les conditions de travail des distributeurs communautaires. D'autres barrières spécifiques au contexte de déploiement ont été identifiées, telles que le choix sous-optimal de la période de déploiement au Burkina Faso ou le manque d'implication des mères/tutrices dans les activités de mobilisation au Tchad.

**Conclusion:**

Dans le contexte de l'adoption croissante de la CPS, cette publication fournit des informations et des recommandations pertinentes pour l'amélioration de la mise en œuvre des programmes de CMS, telles que l'intégration de stratégies répondant aux préoccupations des communautés concernant les effets indésirables des médicaments, les stratégies de mobilisation spécifiques au genre, et/ou l'attention portée aux conditions de travail des distributeurs communautaires. Cette publication souligne également l'importance et l'utilité des recherches en cours sur la qualité du déploiement de la CPS.

## Resumo em português

**Introdução:**

Recomendada desde 2012 pela Organização Mundial de Saúde (OMS), a quimioprevenção sazonal do paludismo (SMC) é uma intervenção de base comunitária para prevenir o paludismo em crianças em zonas da África onde a transmissão do paludismo segue um padrão sazonal. Após a publicação das diretrizes consolidadas da OMS sobre o paludismo, espera-se que a SMC chegue a mais crianças em novas zonas geográficas nos próximos anos. Embora se tenha demonstrado que a SMC reduz a morbilidade e a mortalidade causadas pelo paludismo, há potencial para melhorar a qualidade da implementação da intervenção. Com ajuda dum quadro de padrões de qualidade para a SMC desenvolvido pelo Malaria Consortium, a presente publicação visa compreender melhor a qualidade da implementação da SMC e identificar potenciais barreiras à qualidade da implementação da SMC.

**Métodos:**

Foi realizado um estudo qualitativo baseado na análise secundária dos dados recolhidos após as rondas anuais da SMC implementadas no Burkina Faso e no Chade em 2019. Foi efectuado uma análise temática de 16 discussões de grupos focais realizadas com cuidadores e distribuidores comunitários. Tres padrões do quadro de normas de qualidade para a implementação da SMC forneceu os principais temas de qualidade sob os quais os subtemas identificados foram colocados.

**Resultados:**

Foram identificados oito sub-temas relacionados com padrões de qualidade; planeamento; sensibilização e envolvimento da comunidade; e administração de drogas da SMC. Embora a SMC tenha sido bem aceita pelas comunidades em ambos os contextos, foram identificadas barreiras comuns à implementação duma SMC de qualidade, incluindo: a dificuldade de assegurar o cumprimento do protocolo de administração da SMC; atingir populações móveis; preocupações com reacções adversas aos medicamentos; rumores e preocupações com a SMC; e as condições de trabalho dos distribuidores comunitários. Foram identificados outros obstáculos específicos ao contexto de implantação, tais como a escolha subaproveitada do período de implantação no Burkina Faso ou a falta de envolvimento das cuidadoras femininas nas actividades de mobilização no Chade.

**Conclusão:**

No contexto do aumento da adopção da SMC, esta publicação fornece informações e recomendações relevantes para melhorar a implementação de programas de SMC, tais como estratégias integradoras que abordam as preocupações da comunidade sobre reacções adversas aos medicamentos, estratégias de mobilização específicas de género, e/ou atenção às condições de trabalho dos distribuidores comunitários. Salienta igualmente a importância e a utilidade das investigaçãos em curso sobre a qualidade da implementação da SMC.

## Background

Malaria remains a major contributor to the global burden of disease. In 2020, it was estimated that there were 241 million malaria cases and 627,000 deaths [1]. Sub-Saharan Africa bears almost the entire global malaria burden, with 95% of malaria cases and 96% of malaria deaths. In 2020, children under five accounted for about 80% of all malaria deaths in the region [1]. Sustainable development goal (SDG) 3 targets Good Health and Well-being and aims to reduce the under-five mortality rate to 25 per 1,000 live births by 2030, while aiming to “end the epidemic of malaria” by 2030 [2, 3]. Although considerable progress toward malaria control and elimination has been made in the last decades, it has stalled in the past few years [1]. The target of a 90% reduction in incidence and prevalence of malaria set by the World Health Organization (WHO) Global Technical Strategy for Malaria 2016–2030 is not on track. The 2020 intermediary target of at least 40% reduction of malaria incidence and mortality was not reached [1, 4]. Scale-up of known effective strategies, implemented with adequate quality, is thus necessary.

**Fig. 1:**
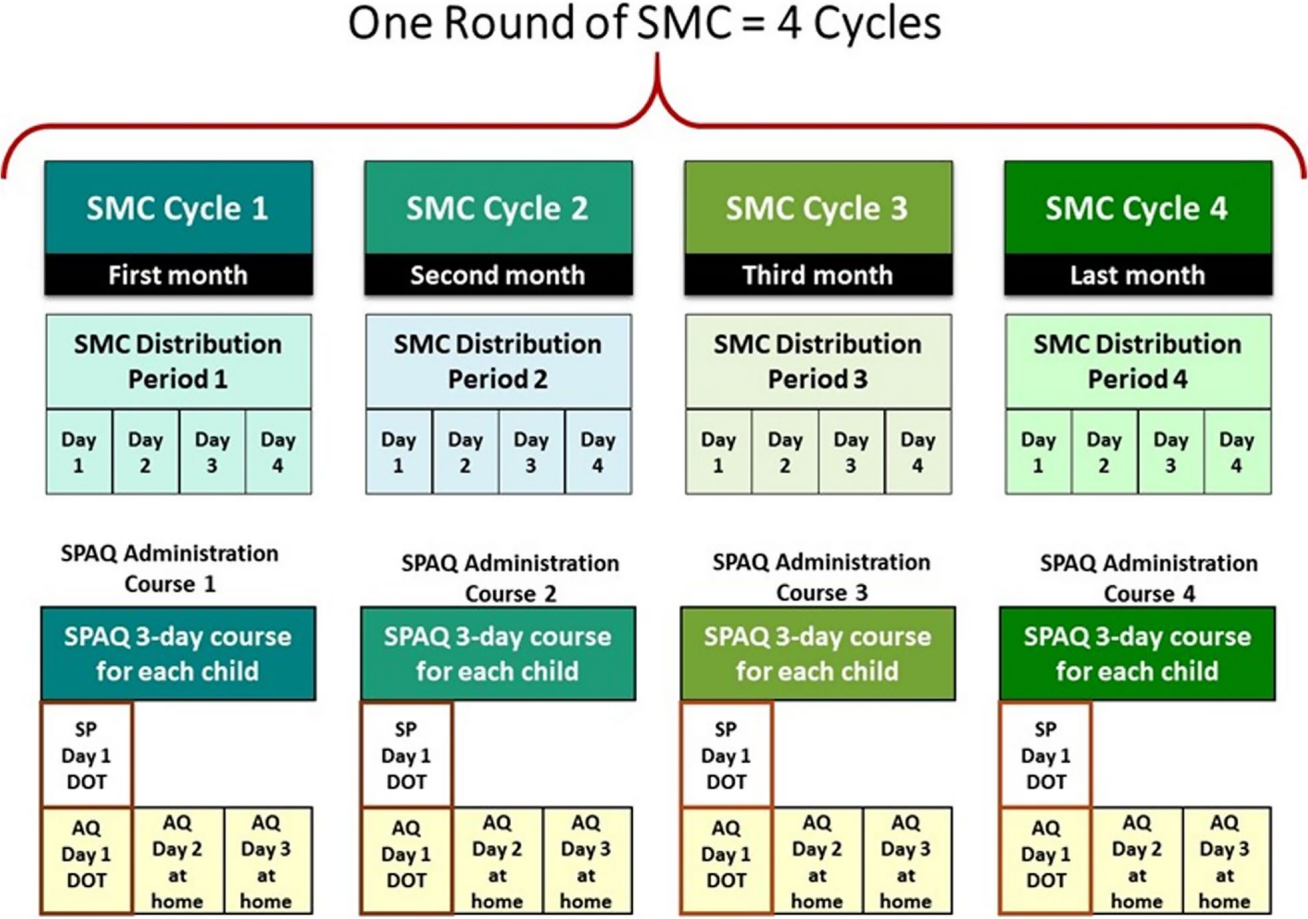
One round of SMC of four cycles. *AQ* amodiaquine, *DOT* directly observed therapy, *SMC* seasonal malaria chemoprevention, *SP* sulfadoxine-pyrimethamine, *SPAQ* sulfadoxine-pyrimethamine plus amodiaquine

One effective malaria prevention strategy for children at risk of malaria is seasonal malaria chemoprevention (SMC). SMC is a form of intermittent preventive treatment recommended by the WHO since 2012 for children aged 3–59 months in areas of high seasonal transmission [5]. SMC consists of the monthly administration of a 3-day course of sulfadoxine-pyrimethamine (SP) and amodiaquine (AQ) to the targeted children during the period of highest malaria transmission. Typically, annual SMC rounds consisting of four monthly distribution cycles are implemented using a door-to-door distribution strategy. During each SMC cycle, the first dose of SP and AQ is administrated as directly observed treatment (DOT) in the presence of a community distributor (CD). The second and third doses of AQ are left to the caregiver to administer daily over the next 2 days [5]. Where Malaria Consortium supports national malaria control programmes for the delivery of SMC, CDs work in pair. They are selected and trained on SMC delivery before the campaign. A monthly refresher training is organized each cycle of the campaign. Throughout the campaigns, engagement activities are organized to support SMC and its delivery. Figure 1 displays a typical SMC round.

There is strong evidence of the effectiveness of SMC in reducing morbidity and mortality from malaria, as well as its cost-effectiveness [6–9]. Recently, a multi-centred observational study showed 88% protective effectiveness of SMC, when delivered on a large scale [6]. Furthermore, SMC along with the malaria vaccine RTS,S/AS01_E_, showed better efficacy when combined than when either intervention was done alone, paving the way for new strategies for malaria prevention [10, 11]. Since 2012, SMC has been increasingly implemented, reaching 33 million children in 13 countries in 2020 [1]. The number of children reached through SMC is expected to continue to increase in the coming years. In 2022, the WHO published consolidated guidelines for malaria, which provide further flexibility for malaria-endemic countries to adapt malaria prevention and control strategies to the local epidemiology. For SMC, the guidelines no longer define geographic restrictions and provide greater flexibility in recognizing aged-based risk [12]. SMC is currently being implemented outside the Sahel region, notably in east and Southern Africa in countries, such as Mozambique and Uganda [13–16].

Although the effectiveness and cost-effectiveness of SMC have been established, the quality of the delivery of SMC can be improved. A mixed-methods study in Burkina Faso, using the Donabedian quality of care framework, judged the overall quality of the campaign conducted in 2017 as acceptable when delivered by community health workers. However, certain elements linked to technicity (for example determination of the patient’s age) and safety (for example hygiene) were scored as low [17]. Another study assessed the implementation fidelity of two SMC rounds conducted in 2014 and 2015 in Burkina Faso. It concluded that less than one third of the children targeted received SMC medicines in all monthly distribution cycles. The study also documented a lack of supplies, training, and financial resources to compensate the CDs [18]. More recently, formative research was conducted in Mali to understand which factors to consider for increasing the quality of SMC interventions. The intervention was well understood and accepted by the community; however, the study reported a low adherence to the SPAQ administration protocol by the caregivers. CDs estimated that three to four of ten children received the full course of SPAQ during one cycle. This low adherence was explained by the fear of side effects, the healthcare expenditures they generate for the households, and conspiracy theories related to them (diminish fertility, spread COVID-19, CDs’ personal gain). The authors stressed the need for improved community engagement around the potential adverse events to SMC medicines [19].

Malaria Consortium, a non-profit organization supporting the implementation of SMC campaigns in Africa, has developed a framework of ten quality standards (QS) for SMC delivery (unpublished). The aim of this framework is to ensure consistency of quality across different locations where the organization supports SMC and define benchmarks against which quality can be assessed. The SMC Quality Workstream of the organization developed it, involving teams responsible for SMC programmes at headquarters and country levels. It was built based on an analysis of SMC operational programming, the available WHO implementation guidelines, and the experience of the Malaria Consortium in supporting national malaria control programmes in implementing SMC campaigns since 2013. The ten QS developed by Malaria Consortium cover the key SMC delivery elements listed in Table 1.

**Table 1:** Malaria Consortium’s ten quality standards for SMC delivery

Quality standard	Definition
Planning and enumeration	Complete an SMC plan 4 months prior to each SMC round which includes enumeration of targeted children, human resource capacity needs including training and supervision, quantification of commodities and expected budget
SPAQ procurement	Procure sufficient quality-assured SPAQ in time to be available at least 2 weeks before the start of the SMC round and ensure its continued availability until the end of the round
Supply management at the country level	Procure and manage the supply and accountability of all SMC commodities and tools before, during and after each cycle
Community engagement	Sensitize and engage with communities before and during each SMC cycle
Training	Provide quality SMC training to trainers, supervisors, health facility workers and community distributors within 1 month of each SMC campaign
Administration of SMC medicines	Deliver a full 3-day course of SPAQ to eligible children each cycle of the SMC round during the period of highest malaria transmission
Referral and case management	Fully assess, treat as required, and record all children referred to the health facility during SMC
Supervision	Supervise, monitor, and report on the performance of each community distributors team once per cycle
Monitoring and evaluation	Conduct routine monitoring and evaluation of SMC inputs, processes, outputs, outcomes, and impact throughout the SMC round
Safeguarding	Ensure the safeguarding of children, caregivers, community members and community distributors during SMC delivery

*SMC* seasonal malaria chemoprevention, *SPAQ* sulfadoxine-pyrimethamine plus amodiaquine

This study aimed to develop a better understanding barriers to quality of SMC delivery across different implementation contexts by adopting a multi-centred approach and focusing on the perspectives of caregivers and community distributors through the cases of SMC campaigns that were conducted in Burkina Faso and Chad in 2019. For the analysis, three of the ten standards of quality service delivery were selected; planning and enumeration, community engagement, administration of SMC medicines. These standards were chosen because they were deemed by the authors to be the most critical in the perspective end users and end implementers of SMC who are in focus in this analysis. Were assessed for each standard; (1) potential barriers to quality delivery of SMC in relation to the QS, and (2) potential similarities and differences between these barriers in the two delivery contexts, Chad and Burkina Faso.

## Methods

### Study design

A secondary thematic analysis based on 16 focus group discussions (FGDs) conducted with caregivers and CDs was performed. The FGDs were gathered during two operational studies conducted after the end of the final round of the SMC campaigns implemented in Burkina Faso and Chad in 2019 [20, 21]. The aim of these studies was to assess the feasibility and the acceptability of the modification of one element of the campaign (addition of a fifth distribution cycle in Burkina Faso instead of the usual four cycles, extension of SMC to children aged 5–10 years old in Chad). The studies had a similar mixed-methods design but did not have quality of SMC delivery for focus. While analysing the FGDs transcripts though, the primary researchers identified quality of SMC delivery as an important theme across them, and thus this additional analysis was deemed valuable. A detailed description of both original study methods can be found in the precedent publications [20, 21].

### Research setting

The national malaria control programmes in Burkina Faso and Chad conducted the SMC campaigns, which are the focus of this paper, with support from Malaria Consortium.

In Burkina Faso, the study took place in the Mangodara health district (South-western region), a rural area where farming is the main economic activity. In Chad, the study was conducted in the Massaguet Health district, in the western part of the country, close to the country’s capital, N’Djamena. Massaguet is mainly a rural area; however, a small proportion of the population lives in urban settlements, and nomadic groups also live in the district. In both districts, the incidence of malaria exceeds the average among the health districts eligible for SMC in both countries, with Mangodara being the district with the highest prevalence in Burkina Faso [22, 23]. Table 2 summarizes the implementation setting for both campaigns.

**Table 2:** Setting of the SMC campaigns conducted in 2019 in Chad and Burkina Faso

	Burkina Faso	Chad
Health district of the campaign	Mangodara (South-western region)	Massaguet (Western region)
Territorial typology	Rural	Mainly rural, some urban settlements, presence of nomadic communities
Prevalence of malaria at country level for children under five	17% [22]	40.9% [23]
Period of the round	June to October 2019 (5 cycles)	July to October (4 cycles)
Target number of eligible children for SMC	30,645 children aged 3–59 months	36,050 children aged 3–59 months
Beginning of SMC campaigns in the district	2014	2015

*SMC* seasonal malaria chemoprevention

### Selection of participants and data collection

**Fig. 2:**
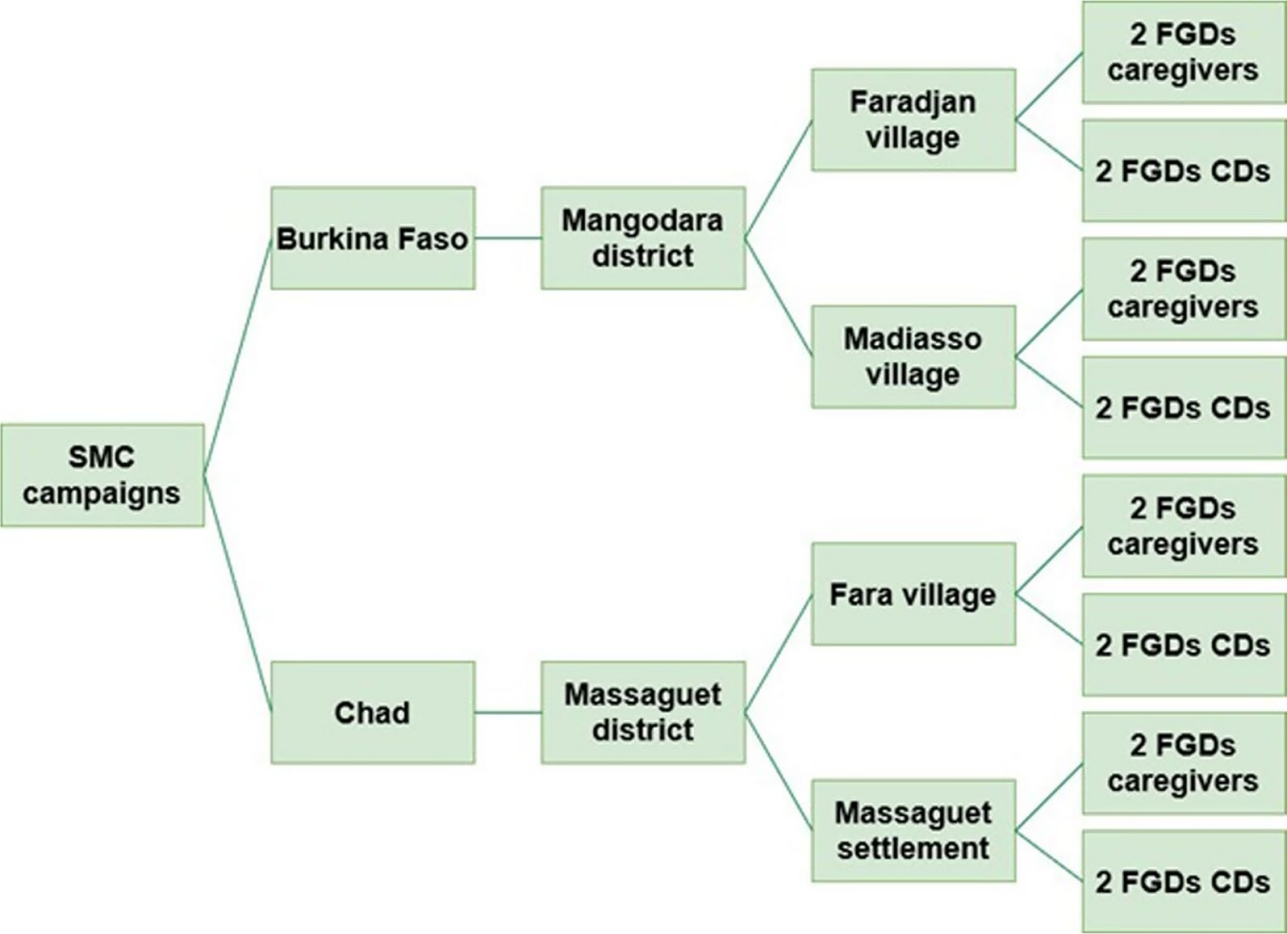
Sampling distribution of the FGDs: geographic locations and participants’ categories. *CD* community distributor, *FGD* focus group discussion, *SMC* seasonal malaria chemoprevention

Sixteen FGDs transcripts were analysed for this paper. In each district, four FGDs were conducted after the final cycle of the SMC round, in two different localities. Two FGDs were conducted with CDs and two FGDs with caregivers for a total of 134 participants (Fig. 2).

The participants’ selection differed slightly between the two countries. In Burkina Faso, the sample selection followed a multi-stage sampling approach. First, two health facilities from the Mangodara district were randomly sampled among the 26 health facilities belonging to the district. One village depending on each randomly selected health facility was then purposively selected (Faradjan and Madiasso). The participants’ selection was purposive. CDs were selected after consultation with the manager of the healthcare facility, while the community leaders of each village were consulted to select the caregivers. Both caregivers and CDs selections were based on their availability and willingness to participate. The FGDs were composed by participants of the same gender to achieve homogeneity in the group and to facilitate the discussion. Due to the lack of female CDs in Madiasso, one FGD was conducted with both male and female CDs.

In Chad, one rural village (Fara) and one urban settlement from the Massaguet district were purposively sampled based on their accessibility by road from the capital Ndjamena. The selection of the CDs and caregivers followed the same principles as in Burkina Faso, with a purposive selection also based on availability and willingness to participate after consultation with the healthcare facilities managers and the village leaders. The FGDs were also composed of participants of the same gender. The choice was made to only conduct FGDs with female caregivers divided into two groups, caregivers younger than 30 years old and caregivers older than 30 years old. The perspective of females, primary caregivers in this context, was deemed to be the most relevant to reaching the objectives of the initial study. In both settings, none of the identified participants refused to take part in the FGDs. Table 3 describes the sample size of the FGDs, their composition and the inclusion criteria.

**Table 3:** Focus group discussions characteristics

	Burkina Faso	Chad
Sample size for each FGD	Caregivers: 10 CDs: 6–10	Caregivers: 8 CDs: 7–8
Inclusion criteria	Caregivers with at least one child aged 3–59 months who participated in SMC in the study district in 2019	Caregivers (aged ≥ 18 years) of children below 10 years old who participated in SMC in 2019
	CDs who participated in 2019 SMC campaign	CDs who participated in 2019 SMC campaign
Composition	Caregivers: 2 FGDs males 2 FGDs females	Caregivers: females only 2 FGDs caregivers > 30 years old 2 FGDs caregivers < 30 years old
	CDs: 2 FGDs males 1 FGD females 1 FGD both males and females	CDs: 2 FGDs males 2 FGDs females
Dates of conduction of the FGDs	18th and 26th of November 2019	6th and 18th of February 2020
Duration of the FGDs	From 45 min to 1 hour and a half. The mean time for the FGDs was 1 hour	

*CD* community distributor, *FGD* focus group discussion, *SMC* seasonal malaria chemoprevention

The FGDs were conducted by research assistants with a background in social sciences who were specifically recruited for the studies and trained by Malaria Consortium research team for conduction of the FGDs before their realization. Malaria Consortium study coordinators for Chad and Burkina Faso directly supervised the realization of the FGDs. Before the FGDs, the topic guides were pilot tested and reviewed. The FGDs took place in the primary healthcare centres of the villages. One research assistant was responsible for leading the FGDs, while another assistant was responsible for taking field notes. The FGDs were conducted in the local languages, Dioula in Burkina Faso, and Chadian Arabic in Chad and digitally recorded. Once transcribed *verbatim,* the transcripts were translated to French and then English. Data saturation was deemed satisfying for the purpose of the original studies, and no additional FGDs were conducted.

### Data analysis

The data were analysed using thematic analysis described by Braun and Clarke [24]. The three QS of the quality framework for SMC delivery provided the overarching themes under which this initial coding was conducted. Although the three QS guided the coding, the process remained flexible, and many codes were generated from the transcripts. Attention was given to generating codes that would capture the specificity of the views of each group (male/female, CDs/caregivers) and the specificity of the local implementation context in Burkina Faso/Chad. The first author conducted the initial coding. A sample of this coding was then shared to three co-authors (two of them familiar with the data as they were involved in the initial studies) who provided feedback on its appropriateness. The codes were then consolidated, and the coding reviewed. Subsequently, a list of eight subthemes fitting under the three QS was established in discussion between the first author and the research teams involved in the initial studies, including Malaria Consortium’s Chad and Burkina Faso study coordinators. The computer-assisted qualitative data analysis software MAXQDA 2022 was used to support the analysis [25].

### Ethics

The original study conducted in Burkina Faso was approved by the Comité Institutionnel de Bioéthique of Centre National de Recherche et de Formation sur le Paludisme (no 2019/000005/MS/SG/INSP/CNRFP/CIB). The original study conducted in Chad was approved by the National BioEthics Committee (no 0173/PR/MESRI/SG/CNBT/2020) and the Liverpool School of Tropical Medicine Research Ethics Committee (Ref: 19–071). All participants provided written consent.

## Results

**Fig. 3:**
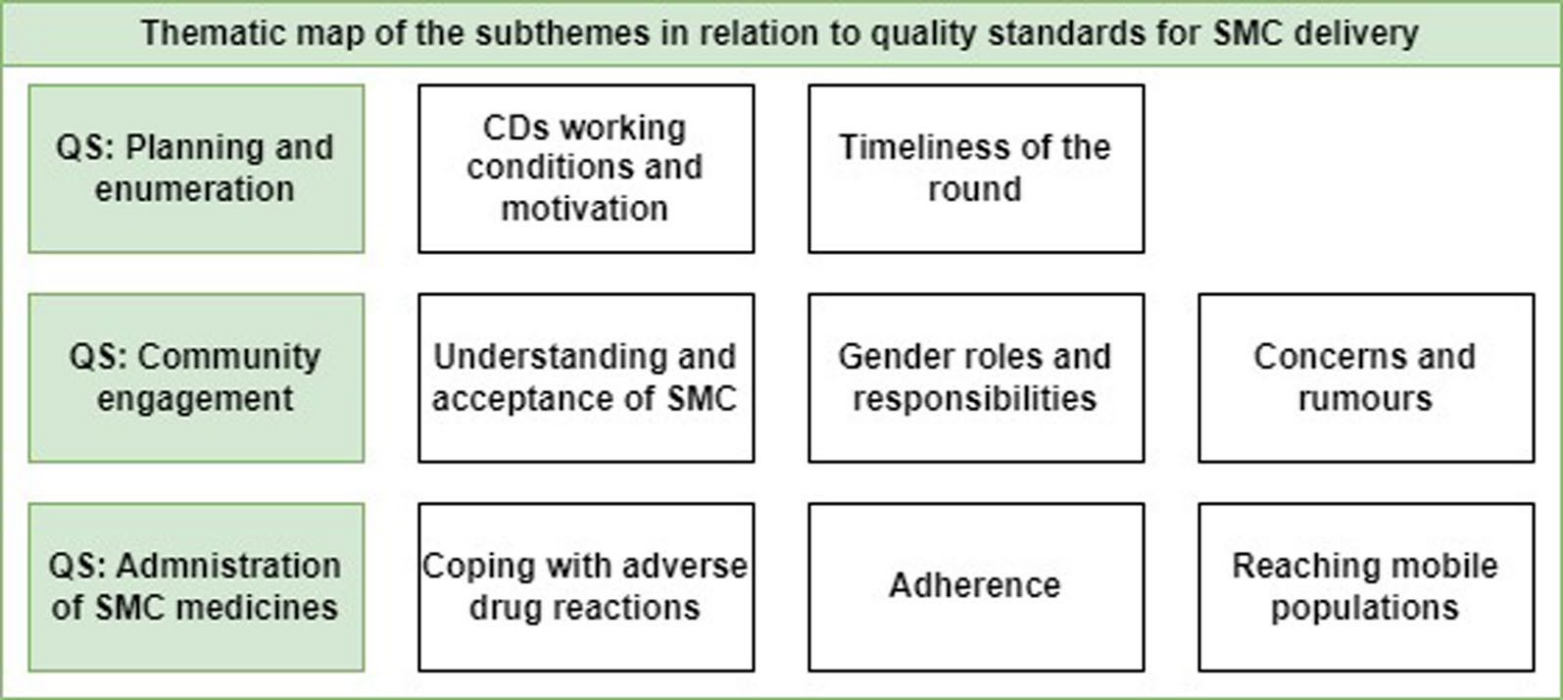
Thematic map of the subthemes in relation to the quality standards for SMC delivery. *CD* community distributor, *QS* quality standard, *SMC* seasonal malaria chemoprevention

Eight subthemes in total were identified relating to the three quality standards for SMC delivery: Planning and enumeration, Community engagement, and Administration of SMC medicines (Fig. 3). Relevant quotes from the FGDs transcripts were chosen to illustrate the findings.

### Quality standard: planning and enumeration

#### Community distributors’ working conditions and motivation

CDs in both Burkina Faso and Chad were generally unsatisfied with their working conditions, as they did not allow them to perform their job as well as they would have liked. Some of them described that they were losing motivation over the years. CDs reported that they started to work with SMC to improve health in their community, that community leaders had chosen them for this role, and that they had the responsibility not to betray their trust. Although CDs valued their work and witnessed their contribution to the better health of the children in their community, they described their working conditions as “discouraging”. The workload is heavy, with an important number of tasks to achieve during the visits, and the number of CDs mobilized for the round was too small in relation to the number of children to cover.

*“There are many children who need to receive SMC. In N'Djamena Fara here, we have 10 community health workers teams for seven villages. It's a lot.” (CD, Chad)*

In Burkina Faso, middle-upper arm circumference measurement was added to the activities during the 2019 campaign as a combined strategy integrating malnutrition screening to SMC distribution. This led to a reported increased workload. CDs also pinpointed the lack of support and encouragement from their supervisors as a demotivating factor. In addition, the concomitance of the SMC round with the intense farming period in Mangadora forced CDs to incur debts for hiring fieldworkers to cover their absence in the field. Other CDs hired motorcycles to be able to cover the distances between the settlements they were responsible for during the distribution. They reported those debts were difficult to reimburse since their incentives were not paid on time, an issue creating conflicts between CDs and their creditors. This was pinpointed as a demotivating factor.

*“(…) when the campaign starts, we are obliged to go into debt to take on contract workers for our fieldwork so that we can run the SMC campaign (…). If there is no contract worker for your fieldwork, it means that this work is wasted.” (CD, Burkina Faso).*

The hardship of the working conditions and the delays in payments were named as reasons to not engage in SMC rounds in the future by some CDs.

*“[…] if it continues with the same conditions of this year, we can say that we shall not be able to continue. Because we told the Chief Nurse that if it carried on like this, there was no need to call us for the coming years.” (CD, Burkina Faso)*

#### Timeliness of the round

In Burkina Faso, the timeliness of the round was discussed intensively by both caregivers and CDs and was not perceived as optimal. This year, the SMC round started in June instead of July in Mangodara health district. This choice was made because the rainfall pattern changed over the years and the rainy season seemed to start earlier in the south-west region. For most caregivers and CDs this change was welcome and appreciated because of the link between the increase in malaria transmission and the beginning of the rainy season and in consequence the need to prevent the transmission early.

However, many caregivers and CDs expressed that an earlier start of the campaign would have been better. By June, the rainy season had already started, and mosquitoes were already present. May was perceived as a more appropriate month to start the campaign.

*“[…] in the month of May already, mosquitoes are out in our community [..] The month of June is good, but the month of May is better.” (CD, Burkina Faso)*

As already pinpointed, the concomitance between the rainy season and the SMC round was discussed as a challenge due to concurring farming activities occurring at the same period. Some CDs reported that they were accused of being lazy by some community members for valuing the additional money they earn working with SMC over their duties as farmers. The tensions occurred especially during June, the beginning of the rainy season, an intense month for farming activities.

*“If they see us with the bags, they start insulting us because it is the rainy season, and we are conducting SMC campaigns. They think we do not like the work in the fields.” (CD, Burkina Faso)*

The timeliness of the round was not discussed in Chad. One of the reasons could be the lesser importance of the farming season for some participants who live in urban settlements.

### Quality standard: community engagement

#### Understanding and acceptance of SMC

Sensitization and community engagement activities were conducted in both countries before and during the SMC rounds. A range of standard activities were conducted from national level media campaigns, stakeholder engagement activities at all levels and community level engagement through villages leaders, religious leaders and mobilization of town criers for each distribution cycle. In both contexts, SMC was widely accepted and appreciated. However, understanding of SMC varied and might have had an impact on the quality delivery of the round in Chad. The preventive role of SMC was not understood by many female caregivers in Chad and might be explained by the fact that they were not directly targeted during community engagement and sensitization activities. Many of the participants thought that SPAQ was a curative treatment for malaria. They expressed incomprehension when CDs administrated SPAQ to their children without performing a clinical examination to assess if the child would need the treatment. Other caregivers did not understand why the SMC campaign targeted only healthy children, leaving sick children without treatment.

*“In some cases, they do not give to a child who is already suffering. So, we don't know why that.” (Caregiver, Chad)*

The misunderstanding of the preventive principle of SMC in Chad may have led to misuse of the SMC medicine by caregivers. Some reported keeping the tablets for later use to treat their children or themselves when ill. One CD also reported taking the drug himself as a treatment for malaria when feeling sick.

In Burkina Faso, the principle of SMC as a strategy to prevent malaria during the rainy season was well understood by caregivers. There was also a common understanding that SPAQ would be ineffective to treat a child who already had malaria.

#### Gender roles and responsibilities

A transversal element across the study settings was the recognition of mothers as primarily responsible for administering SMC. In contrast, fathers were described as decision-makers for accepting SMC or not and could have a supportive role in reminding the mothers to administer the second and the third doses.

CDs and caregivers from Chad reported a lack of involvement of mothers and women in engagement activities conducted around SMC which might have led to a lack of understanding of SMC principles by the mothers. Mothers depended on the mobilization of their husbands to be informed about the organization of the campaign. This was perceived as ineffective by caregivers, and both CDs and caregivers stressed that community engagement activities targeting women and the recruitment of females in the mobilization teams would enhance SMC’s understanding and adherence.

*“Awareness there, we want it to be more for women. Because the husband will not be able to bring awareness to the woman. If you, a woman, go to another woman, you can sensitize her. She will listen to your words.” (Caregiver, Chad)*

*“In my opinion, in each district there should be some leader-women so that they can sensitize themselves their friends, sisters and parents.” (CD, Chad)*

In Burkina Faso, husbands’ engagement was considered as pivotal when it comes to achieving adherence by following up on AQ administration.

*“After the administration of the first dose, if there is no follow-up by the head of the family, the remaining doses will never be administered.” (CD, Burkina Faso)*

Some male caregivers were reported to prioritize fieldwork instead of their children’s health by going to the field on distribution days and not waiting for the CDs’ arrival. Several participating caregivers expressed their disapproval of this attitude, which demonstrates a lack of “care” of those heads of families for their children's health.

#### Concerns and rumours

In both settings, caregivers reported rumours around concerns circulating in their community about SPAQ, which might have influenced the caregivers’ respect of the SPAQ administration protocol and impacted adherence. In Chad, some community members were reported expressing concerns about the provenance of the pills and the way they were manufactured.

*“They say white people are cheating on us. “Were you present when they manufactured the drug?” We tell them we weren't there when the drugs were made. But, from what is written, we know that it is a drug that treats malaria.” (CD, Chad)*

Some CDs themselves expressed the same concerns about the provenance of the pills. Other community members reported thinking that the SMC drugs distributed were expired. Participants also reported the existence of a recurring talk circulating in the community that SMC is used to make the population sterile.

In Burkina Faso, many respondents reported that some people believed SMC was administrated purposively to make children sick. One way for the government to earn money from the payments received by the healthcare centres treating diseases induced by SMC.

*“Some people say that those who give the medicine do it so that the children will manifest the illness and we will have to go to the healthcare centre to treat them. (…). They think that what the government is doing is just to sell the drugs.” (Caregiver, Burkina Faso)*

### Quality standard: administration of SMC medicines

#### Coping with adverse drug reactions (ADR)

Across the data, the ADRs due to SPAQ administration were extensively discussed by the participants. Some caregivers reported that most children experienced ADRs and that many children suffering from ADRs were brought the healthcare centres during the distribution period. The majority of the ADRs mentioned, including fever, vomiting, diarrhoea, skin rash or weakness, were mild, and no severe ADRs were reported.

ADRs made administering the second and third doses of SMC difficult for the caregivers, seeing their children who previously were healthy suffer due to the drug. Caregivers in Burkina Faso discussed at length the difficulty provoked by ADRs. Some caregivers mentioned the “courage” it took to administer the full 3-day course, while others mentioned the “doubt” the ADRs instilled in the caregiver’s mind when SMC, supposed to bring a better health status to their children, made them sick.

*“At the beginning of the distribution of the drugs in June, my child took the first dose, it made him/her suffer. In fact, he/she vomited and had diarrhoea and fever too. But I continued to give the rest, and it felt good. So I can say that at the first dose, if you are not courageous enough, you cannot continue. Because it scares you. It knocked the child down (laughs), it knocked the child down.” (Caregiver, Burkina Faso)*

The participating caregivers reported giving the whole course of SMC medicines despite ADRs because they believed in the efficacy of SMC and its long-term benefits on their children’s health. Nevertheless, caregivers and CDs recognized that some other caregivers in the community refused to administer the second and third doses of AQ when their children developed ADRs following the first doses of SP and AQ.

In both Burkina Faso and Chad, some CDs also described the ADRs as a complication in their work and indicated that ADRs negatively affected the acceptance of SMC by the population. One caregiver from Burkina Faso proposed community engagement activities targeting ADRs, which could increase the acceptance of SMC and help caregivers to understand and better address ADRs.

*“If they could find a way to raise awareness in order to draw people's attention to malaria prevention drugs, it would increase people's knowledge. For example, they can talk about the difficulties that can arise after taking the drugs. It sometimes confuses some people. But if information is given to them, it will help them know that these are the effects of the medicine's action. For me, it can help people to better understand and accept the effects.” (Caregiver, Burkina Faso)*

In Chad, CDs reported that ADRs generated expenditures for the caregivers. Some caregivers reported to them that treatment provided by the HCF for children with ADRs was charged. This negatively impacted the acceptance of SMC by some community members according to the CDs.

#### Adherence

In both Chad and Burkina Faso, respondent caregivers understood how to give day two and three AQ doses overall and respected the recommendations given by the CDs. However, some reported neighbours and other community members throwing doses away or keeping them for future use. The concern about some caregivers not administrating the remaining doses was also reported by several CDs.

*“The SMC campaign ended in the 10th month. But today, you will find the SMC tablets if you rummage through some women's wardrobes. I assure you! Let's not talk about those who put everything in the trash.” (CD, Chad)*

*“There are some people, when the CDs give them the medicine, they do not give it to their children. The mother can throw it in the toilet but how would you know whether she gave it or not.” (Caregiver, Burkina Faso)*

CDs highlighted strategies to follow up on adherence to the second and third doses of AQ. Some CDs reported controlling the administration of the remaining doses by asking caregivers to show them the empty blister packs the day after their visit and encourage the caregiver to administer the medicines if not done. Other CDs asked caregivers if they administrated the remaining doses as indicated while visiting the households during the next distribution cycle.

The lack of control over adherence to the doses administered on day 2 and 3 is considered a problem by some CDs in both settings. Among their suggestions for improvement was including systematic door-to-door follow-up visits in the cycles to all households whose children were targeted by the campaign. This follow-up would be done once the 3 days of SMC medicine is over, to ensure the administration of the second and third doses was respected by the caregivers.

*“We think that some mothers follow the instructions and others throw out the window as soon as we turn our backs, it is difficult to make a return after door to door to check since we only have 3 days for SMC administration. For a follow-up, the number of days must be increased, so after 3 days, we come back to check if the mothers have done the work, ask them about the reasons why they did not give the tablets.” (CD, Chad)*

*“If they could modify the campaign so that the CDs could go at least twice to each courtyard, it would reduce the problem [of adherence]. The first dose is well administered, but the other doses are not always administered by the mothers.” (CD, Burkina Faso)*

CDs from Burkina Faso also discussed that a one-dose regimen would benefit SMC programmes, since CDs would have direct control over the administration of the pill.

#### Reaching mobile populations

Overall, CDs in both settings perceived that most of the eligible children in their area of distribution were reached by SMC. An exception reported were children from nomadic communities and children living in areas where populations move across borders. In Burkina Faso, CDs reported difficulties in ensuring SMC coverage in the population moving back and forth across the border to Côte d’Ivoire during the first cycle of the campaign. When the target population was estimated before SMC delivery, mobile populations were not included and insufficient SPAQ was allocated. In the case of Chad, CDs reported that the pastoral nomadic communities of the area did not benefit from SMC that year.

## Discussion

This paper aimed to better understand the quality of delivery of SMC after several years of implementation at scale in Sahel and identify common and context-specific potential barriers to quality delivery through the cases of two SMC rounds conducted in Chad and Burkina Faso. Although SMC was well accepted by the communities in both study settings, several barriers to the quality delivery of SMC were identified, including difficulty ensuring adherence to the SPAQ administration protocol, challenges reaching mobile populations, concerns around the adverse drug reactions, circulating of rumours, and the difficult working conditions of the CDs. Some barriers were context-specific, such as the suboptimal timeliness of the round in Burkina Faso or the lack of involvement of female caregivers in mobilization activities around SMC in Chad.

Adherence to the SPAQ administration protocol was reported as not optimal by participants in both contexts. CDs stressed that better monitoring of adherence to SMC medicines was needed. Suboptimal adherence to SPAQ in other SMC campaigns has been found in several studies in Burkina Faso in other contexts [18, 19, 26–28]. Earlier studies explain this lack of adherence due to the form and taste of the medication, making the administration difficult [18, 19], since, child-friendly formulations of SPAQ have been developed and were used during the SMC campaigns in Chad and Burkina Faso in 2019. Other explanations for suboptimal adherence included negligence or omission from the caregiver [28], or the fear of ADRs [19, 26]. In both contexts, there were extensive concerns expressed around the ADRs caused by SMC medicines; these concerns could have negatively impacted adherence, as this was also observed in an SMC round in Mali [19]. Moreover, the lack of understanding of the prevention principle of SMC by some caregivers could lead to the use of AQ for other purposes, such as sparing the pills for later use as a curative treatment, and could also have impacted adherence. The monitoring and evaluation report for the two SMC rounds which are the focus of this paper estimated through representative household surveys that administration for the second and third doses was at least 98.0% and 97.8% per cycle in Burkina Faso and Chad, respectively [29]. This does not align with the perception of CDs and caregivers and highlights potential challenges of adherence assessment. SPAQ adherence assessment methods are based on self-reports from caregivers and tend to overestimate adherence because of recall bias and social desirability bias [6, 30]. In Niger, a recent estimation of adherence to the SPAQ administration protocol conducted through household surveys concluded that 84% completed the protocol [31]. However, another study also conducted in Niger estimated adherence through the pharmacokinetic measuring of AQ concentration in blood samples found that adherence to the SPAQ administration protocol was as low as 20% for each cycle [30]. Lack of adherence can compromise the protective effects of SPAQ because of the suboptimal drug concentration and could contribute eventually to the emergence of resistance [32, 33]. Continuous efforts to increase awareness about SMC should be made by implementers in terms of community engagement and sensitization such as; the involvement of a broad range of community leaders in the microplanning of the rounds; community engagement activities throughout the year and not just at the time of the campaign; tailor community engagement activities to the delivery context; and intensification of the sensitization at least 1 month before the round start; door to door sensitization a few days before the distribution start; systematic community feedback collection after the cycles and their integration into the next cycle/round [34, 35]. These activities would require resources in often resource dire contexts but would greatly enhance understanding and adherence to SMC, key aspects to its delivery. They also could be integrated to other activities that are or need to be conducted in the health districts.

Another finding of this analysis is the concerns expressed by the participants around SPAQ’s ADRs and the lack of strategies to address them. Caregivers expressed the view that SPAQ administration could be difficult, as it could potentially harm their previously healthy child. There is conflicting evidence about ADR frequency linked to SPAQ [6, 36, 37]. However, the safety of SPAQ is well-established and the occurrence of major severe ADRs is rare [6, 36]. In our settings, no participant reported symptoms that could be interpreted as severe ADRs among the beneficiaries. However, the occurrence of mild ADRs such as vomiting, diarrhoea, fever, or skin reactions, was widely reported. The lack of community engagement around ADRs was already observed during SMC rounds conducted in Mali and Burkina Faso and SPAQ’s ADRs were already pinpointed as a factor affecting the acceptance of SMC by several studies [18, 19, 26, 27]. Integrating messages about potential ADRs and their management in community engagement activities before and during distribution were among the factors contributing to the success of neglected tropical diseases (NTDs) preventive campaigns relying on CDs [38, 39] and could be adopted for SMC.

In both settings, negative rumours regarding SMC were spread within the communities. In Burkina Faso, possibly in link with the concerns expressed around the ADRs, the main rumour was that SMC was given to children to make them purposively sick. In Chad, SMC was said to impact fertility. These types of rumours were previously identified in a study conducted in Mali [19]. There is strong evidence of the negative impact of rumours on the success of mass drug administration campaigns for NTDs relying on CDs. Circulating rumours negatively influenced the decision of the beneficiaries to accept and adhere to the proposed treatment [40].

There was a lack of gender-specific mobilization strategies around SMC. In Chad, mobilization activities around SMC focused on community leaders and engaged more male caregivers than female caregivers. In Burkina Faso, some male caregivers were reported not being sensitized enough about the importance of SMC and tended to prioritize fieldwork. The low involvement of male caregivers during SMC rounds has been documented in a previous study conducted in Burkina Faso [18]. In our results, it appears that gender may play a role when it comes to attitudes and responsibilities toward SMC. Female caregivers are most often responsible for SMC administration to children, while male caregivers are decision-makers when it comes to accepting SMC and play a role as facilitators for SMC administration by reminding their partner to give the remaining doses if forgotten. Gender in relation to malaria remains an understudied field, and there are no publications to the authors’ knowledge regarding SMC in relation to gender [41]. However, the power imbalance in decision-making regarding malaria treatment and prevention in favour of males within the household has been documented. This power imbalance is even more critical within agricultural communities [42, 43]. Nonetheless, studies also suggest that women might be more prone to adopt and sustain malaria prevention measures than men [44, 45]. The lead-mother model strategy in Nigeria is an intervention that is seen to have potential in improving the adoption of healthy behaviours by caregivers during SMC campaigns and could be tailored to other contexts [35].

This paper highlights CDs perception of their working conditions as difficult and the potential impact on their motivation. The high workload, the accumulation of tasks, the out-of-pocket expenditures related to the campaign, delays in the payment of the incentives, or supervision experienced as unsupportive were several factors that impacted CDs motivation. However, their commitment toward the community was a motivating factor despite the frustrations. Nonetheless, several CDs were considering not taking part in SMC delivery in the future if their working condition were not revised. The CD expressed difficulties due to their workload was documented in another study conducted in Burkina Faso, where CDs had to compromise on hygiene or the time dedicated to SMC information provision to caregivers [17]. Good working conditions and a high level of motivation are essential factors for retaining CDs over the years. The repetition of the campaign and annual refresher trainings has been shown to allow them to improve task management and provide better care [46].

Difficulties were noticed in the current SMC delivery strategies to reach mobile populations, resulting in a perceived inadequate or inexistent SMC coverage for these communities. SMC coverage among Chadian nomadic populations has been reported as low despite their quite high awareness, leading to a reported feeling of exclusion and discrimination [47]. Flexible SMC delivery, tailored to the local context, should be integrated in the early stages of the intervention planning for reaching mobile populations. Studies are ongoing testing these intervention adaptations.

Finally, the study also highlights how timeliness of SMC campaigns should be carefully determined, especially in a context where climate change is expected to further impact the seasonality of malaria transmission, and this is especially valid in the Sahel [48].

This analysis has important strengths. It was supported by a specific SMC quality standards framework, which provided a comprehensive list of the key delivery elements to support analysis, of which three QSs were selected and used. Moreover, the multi-setting design of the study supports the possible generalizability of some findings to other implementation settings. Context-specific findings might be transferable within other implementation sites in Chad or Burkina Faso (or elsewhere), but a complete situational analysis of the context similarities with Mangodara and Massaguet districts should be done before.

This paper also has limitations. Foremost, it is a secondary analysis of qualitative data, which is appropriate for answering additional research questions a posteriori from previously gathered material, but the primary data were not initially collected to answer our aim. This limitation is mitigated by the fact that the primary researchers who identified the density of elements linked to quality while conducting the initial analysis on the FGDs are co-authors of this paper. An additional limitation is that we assessed only three among ten QS, and more evidence is needed in applying the full framework to the intervention. Another limitation was the risk of desirability bias during the FGDs, leading to possible understatements when criticizing the SMC campaigns, reporting believing rumours, or not having administered the SMC medicines. To minimize this risk of bias, the research assistants always clarified their, and the research, impartiality from the SMC programmes and SMC delivery. The lack of perspective of male caregivers from Chad also is a limitation, particularly when interpreting the results and discussion regarding gender roles and responsibilities. Additionally, the primary analyst of this paper was not involved in the data collection which may have limited his understanding of the context. However, the analysis was supported by researchers directly involved in the data collection. They also provided their support through validating the thematic map, contributed to contextualizing the results, and reviewed the manuscript. Finally, no additional information sources besides the FGDs transcripts, such as logbooks or observation data, were used in this study and no triangulation was conducted.

### Implications and recommendations

This paper has relevant implications for the future design of SMC campaigns. The planning of SMC campaigns should consider integrating strategies addressing communities’ concerns around ADRs, including messages about their low occurrence and relative mildness, and their management before and during distribution; strategies to identify circulating negative rumours around SMC and mitigate them through behavioural change communications such as community dialogue; specific community mobilization strategies that would target both female and male caregivers; specific and flexible distribution strategies targeting mobile populations; and improved working condition for the CDs. Moreover, further exploration of the discrepancies between the qualitative results presented here, and the high adherence found by representative surveys would be a valuable next step. Eventually, developing single dose SMC regimens provoking fewer ADRs would be ideal. Finally, the results also highlight the need for further research looking at SMC through a gender lens.

## Conclusion

SMC is expected to be increasingly implemented in the coming years and reach significantly more children in countries where it is already adopted and in new regions. In this context, improved quality assurance of SMC campaigns will be essential. Based on the distributions conducted in Chad and Burkina Faso, and with the support of an SMC quality delivery framework, this paper highlights several areas for intervention improvement in relation to the planning, community engagement and sensitization around SMC, and the administration of SMC medicines. It also pinpoints the need for ongoing and robust research on the quality of SMC delivery, especially given the increased adoption of the strategy.

## Data Availability

The FGDs used and analysed during the study are available from the corresponding author upon reasonable request.

## Abbreviations

ADR: Adverse drug reaction
CD: Community distributor
CHW: Community health worker
DOT: Directly observed therapy
FGD: Focus group discussion
HCF: Healthcare facility
NTD: Neglected tropical disease
QS: Quality standard
SDG: Sustainable development goal
SMC: Seasonal malaria chemoprevention
SPAQ: Sulfadoxine-pyrimethamine amodiaquine
WHO: World Health Organization
